# Molecular Imprinted ZnS Quantum Dots-Based Sensor for Selective Sulfanilamide Detection

**DOI:** 10.3390/polym14173540

**Published:** 2022-08-29

**Authors:** Xin Zhang, Pengfei Jiao, Yihan Ma, Yuping Wei

**Affiliations:** 1 School of Life Science and Agricultural Engineering, Nanyang Normal University, Nanyang 473061, China; 2 Research Center of Henan Provincial Agricultural Biomass Resource Engineering and Technology, Nanyang 473061, China

**Keywords:** molecular imprinted polymers, zinc sulfide quantum dots, fluorescence sensor, sulfanilamide

## Abstract

Combining molecular imprinted polymers and water-soluble manganese-doped zinc sulfide quantum dots (Mn^2+^: ZnS QDs), a new molecule imprinted polymers-based fluorescence sensor was designed. The molecule imprinted quantum dots (MIP@QDs) were constructed by coating molecular imprinted polymers layer on the surface of ZnS: Mn^2+^ QDs using the surface molecular imprinting technology. The developed MIP@QDs-based sensor was used for rapid and selective fluorescence sensing of sulfanilamide in water samples. The binding experiments showed that the MIP@QDs has rapid fluorescent responses, which are highly selective of and sensitive to the detection of sulfanilamide. The respond time of the MIP@QDs was 5 min, and the imprinting factor was 14.8. Under optimal conditions, the developed MIP@QDs-based sensor shows a good linearity (R^2^ = 0.9916) over a sulfanilamide concentration range from 2.90 × 10^−8^ to 2.90 × 10^−6^ mol L^−1^, with a detection limit of 3.23 × 10^−9^ mol L^−1^. Furthermore, the proposed MIP@QDs-based sensor was applied to the determination of sulfanilamide in real samples, with recoveries of 96.80%–104.33%, exhibiting good recyclability and stability. Experimental results showed that the prepared MIP@QDs has the potential to serve as a selective and sensitive sensor for the fluorescence sensing of sulfonamides in water samples.

## 1. Introduction

Sulfonamides are classes of synthetic antibiotics, and they are extensively exploited as antibacterial agents in humans, animals and aquaculture [1]. Sulfonamides have the advantages of a broad antibacterial spectrum, good curative effect, low price, and stable structure [2]. However, excessive use of sulfonamides may result in drug residues in foodstuffs and the environment, posing a serious threat to human health and environmental safety [3,4]. Moreover, veterinary use of sulfonamides antibiotics can migrate into water and soil through their metabolites or degradation, polluting the surface run-off and earth [5,6]. At present, there are various techniques are used for routine detection of sulfonamides, such as high-performance liquid chromatography [7,8], electrochemical determination [9,10,11,12], immunosorbent assay [13,14,15], chemiluminescence [16,17], solid-phase extraction [18,19,20,21], capillary electrophoresis [22], and chemo sensor [23,24,25,26]. Although these methods can accurately detect sulfonamides in samples, they require expensive and sophisticated instruments and a complex or time-consuming sample pretreatment process. Therefore, it is of great significance to develop a convenient, low-cost, rapid, and highly selective strategy for the analysis of sulfonamides.

Quantum dots (QDs) are a kind of nanocrystals made of semiconductor materials with electrons and holes that are quantum-confined [27]. QDs have unique physical and optical properties, such as quantum size effect, good optical stability, high luminescence efficiency, size-dependent emission band, high surface volume ratio and biocompatibility, and are widely used in biological analysis and sensors [28,29]. Various works about QDs have mainly focused on the development of novel and potential sensors based on the fluorescence quenching of quantum dots [30,31,32,33]. Molecular imprinting technology copolymerizes the functional monomer with a given molecular by cross-linking and then removes the template molecule to form molecularly imprinted polymers (MIPs) [34]. The binding sites on the prepared MIPs are complementary to the target molecule in their structure, shape, and size [35]. The prepared MIPs have the advantages of strong structure stability, low cost, ease of preparation, and high selectivity [36,37,38,39,40]. This advanced and elegant technology can construct the MIP layer on the surface of the QDs’ matrix materials, which endows the QDs with high selectivity to the target analyte. In this way, a novel selective fluorescence sensor based on surface molecular imprinted QDs can be fabricated and used for rapid analysis of the target molecules [41,42].

In this work, a novel and facile molecularly imprinted quantum dot sensor was developed for rapid fluorescence analysis of the sulfanilamide (SAM) in water samples. Mn^2+^-doped ZnS nanocrystals’ QDs were prepared and used as matrix materials. Mercaptoethylamine was used to modify the Mn^2+^: ZnS QDs to improve their optical stability and biocompatibility. The polymer layer of SAM was imprinted on the surface of the QDs, via surface molecular imprinting technology, to form a “core–shell” structure. In this way, the imprinting efficiency of the polymer and the mass transfer efficiency and binding ability of the template molecule can be effectively improved. Therefore, the SAM MIP@QDs-based sensor with the fluorescence characteristics of quantum dots and the biomolecular recognition of MIPs was successfully prepared. The constructed MIP@QDs not only retain the traditional advantages of molecularly imprinted polymers such as physical stability, selectivity, low cost, and flexible operating conditions, but also have the advantages of a fast mass transfer rate, easy elution/adsorption, and a lower detection limit [43,44]. When the MIPs layer on the MIP@QDs selectively captures the target molecule, a non-radiative transition will be generated between the bound target molecule and the quantum dot [45]. The SAM molecules bound to the MIPs will cause a significant decrease in the fluorescence of the quantum dots, which will transform the molecular recognition signal into a photoelectric signal analysis. Based on this, the prepared MIP@QDs can serve as a fluorescence sensor for the highly sensitive and selective analysis of target molecules. The study also providing a new tool and method for the rapid detection of environmentally harmful chemicals.

## 2. Materials and Methods

### 2.1. Materials and Instrumentation

Sulfanilamide (SAM), sulfanilic acid (SAA), sulfamethoxazole (SMX), ZnSO_4_·7H_2_O, Na_2_S·9H_2_O, 2,2′-azobis(2-methylpropionitrile) (AIBN), and MnCl_2_·4H_2_O were purchased from Macklin Biochemical Co. Ltd. (Shanghai, China). Ethylene glycol dimethyl acrylate (EGDMA), methacrylic acid (MAA), and mercaptoethylamine (MEA) were obtained from J&K Scientific (Beijing, China). 

The fluorescence spectra were measured by a FL-970 spectrofluorometer (Techcomp, Shanghai, China). The morphology of the prepared QDs and MIPs were analyzed using a JEM-2100F transmission electron microscope (TEM, JEOL, Tokyo, Japan). UV spectra were determined on a Specord 210 plus spectrophotometer (Jena, Germany).

### 2.2. Synthesis of MEA-Modified Mn^2+^: ZnS QDs

MEA-modified Mn^2+^: ZnS QDs was prepared by chemical precipitation method [46]. Briefly, 0.5 mmol ZnSO_4_·7H_2_O, 0.04 mmol MnCl_2_·4H_2_O were added in 50 mL ultrapure water. After stirring under nitrogen protection for 1.0 h, 8 mL of 0.5 mol Na_2_S·9H_2_O aqueous solution were dropwise added. Kept stirring for another 12 h under dark conditions, 5 mL ethanol solution of MEA (0.80 mmol) was added and stirring reaction for 20 h. The obtained of MEA-modified Mn^2+^: ZnS QDs were collected by centrifuging.

### 2.3. Preparation of MIP@QDs Nanocomposites

The SAM MIPs layer was coated on the surface of MEA-modified Mn^2+^: ZnS QDs by surface imprinting technique to fabricate MIP@QDs nanocomposites. Firstly, 50 mg SAM and 500 μL MAA were added in 15 mL methanol solution and stirred for 30 min. Secondly, 100 mg MEA-modified Mn^2+^: ZnS QDs and 100 μL EGDMA were added and ultrasonically dispersed for 30 min. Thirdly, 20 mg of AIBN was added and Uv initiated polymerization under 360 nm for 12 h in the dark. Finally, centrifugal collection product was washed with methyl alcohol. As a control, non-imprinted polymers (NIPs) were synthesized by the same methods but absent of SAM. The prepared polymers were repeatedly eluted with a mixture solution of hydrochloric acid and methanol (1:9, *v*:*v*) to remove the SAM molecule. The final product is freeze-dried in vacuum.

### 2.4. Effect of pH Value of Solutions 

The effects of different pH values in solution on fluorescence characteristic of MIP@QDs was studied. Then, 10.0 mg of the prepared polymers were ultrasonically dispersed in 10.0 mL of different pH-value buffer solutions (pH 4.0–10.0). A certain amount of SAM was dissolved in the above solution, and the fluorescence changes in the sample solution were recorded at 312 nm by FL970 fluorescence spectrofluorometer (Techcomp, Shanghai, China).

### 2.5. Fluorescence Measurement of SAM

The prepared MIP and NIP polymers were ultrasonically dispersed in the buffer solution (pH = 8.5). A series of SAM standard solutions were prepared and sequentially added in the above solutions. The mixture was diluted to 10 mL, and the concentration range of SAM was from 2.9 × 10^−8^ to 2.90 × 10^−6^ mol L^−1^. After being incubated for 5 min, the fluorescence emission intensity of the solution was recorded. The SAM concentration in the sample was calculated by Stern–Volmer equation:

(1) F0F=1+KSV[C]

*F*_0_ is the initial fluorescence intensity of the samples without SAM molecule; *F* is the fluorescence intensity of sample with SAM molecule; *K_SV_* is the quenching constant; and *C* is the concentration of the SAM in the sample. Additionally, the quenching degree of MIP@QDs by SAM is denoted by △*F*, △*F = F_0_/F* − 1.

### 2.6. Selectivity Experiments

Sulfanilic acid, 4-ethylaniline, and benzenesulfonic acid were used as references to carry out the selectivity experiments. The MIP- and NIP@QDs were added to SAM or reference solution with the different concentration. After being incubated for 5 min, the fluorescence changes in the sample solution were detected by the spectrofluorometer. The selectivity of the MIP-based sensor for SAM was evaluated using the imprinting factor (*IF*):

(2) IF=KSV,MIPKSV,NIP

where *K_SV,MIP_* and *Ks_V,NIP_* are the quenching constants for the MIP- and NIP@QDs with target molecule, respectively.

### 2.7. Real Sample Analysis

In order to investigate the practicability of the developed sensor, the prepared MIP@QDs were used to analysis the environmental water samples. The samples were collected from rivers and lakes (Nanyang, China). All the samples were filtered with a membrane of 0.4 μm and stored in 4 °C. Then, 3 mL of the samples was diluted to 10 mL with buffer (pH 8.5), the MIP@QDs were added. and they were ultrasonically dispersed in the samples. After being incubated for 5 min, the fluorescence intensity of the MIP@QDs was detected and the concentrations of SAM in the sample were calculated.

## 3. Results

### 3.1. Fabrication of MIP@QDs-Based Sensor

**Figure 1:**
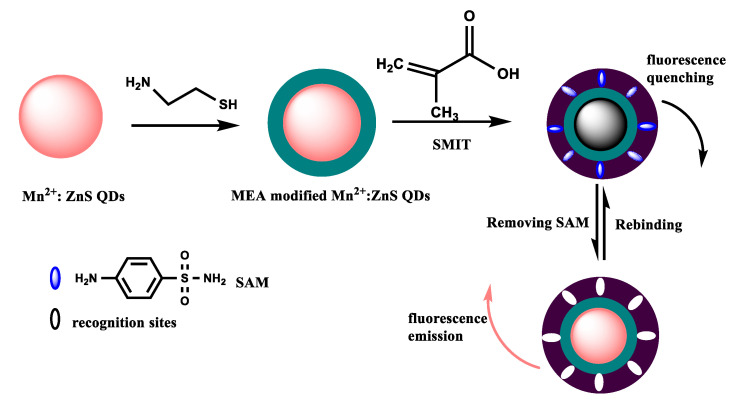
Schematic of the preparation of the MIP@QDs-based sensor. SMIT: surface molecular imprinting technique; SAM: sulfanilamide; MEA: mercaptoethylamine.

The synthetic process of the MIP@QDs-based sensor was illustrated in Figure 1. In the first step, the prepared Mn^2+^: ZnS QDs was modified by MEA via ligand competition. This process can enhance the optical stability and improve the dispersion of Mn^2+^: ZnS QDs in the aqueous phase. In addition, the modified MEA on the QDs facilitates the binding of the function monomer to the template molecule, making it easier to form molecular-recognition sites. In the second step, MAA used as a monomer interacted with the SAM molecule via a hydrogen bond. The MEA-capped Mn^2+^: ZnS QDs was used as substrate materials; EGDMA and 2,2′-azobis(2-methylpropionitrile) were selected as the crosslinker and initiator, respectively. The SAM MIPs layer was polymerized on the surface of QDs through the surface molecular imprinting process. The MIPs layer on the QDs makes it possible to selectively recognize the target molecule and avoid the fluorescence quenching caused by other interfering substances from contacting the QDs. The prepared MIP@QDs were eluted to remove the SAM molecules, and the binding sites complementary to the SAM molecules are formed on the MIPs layer. This preparation process of MIP@QDs was straightforward, time-saving, and cost-effective. The resulting MIP@QDs not only possesses the highly selective of MIPs but also inherits the optical characteristics of ZnS QDs. The prepared MIP@QDs could be used as an ideal sensing matrix material to fabricate an efficient sensor that is highly sensitive and selective for the target molecule.

### 3.2. Characterization

**Figure 2:**
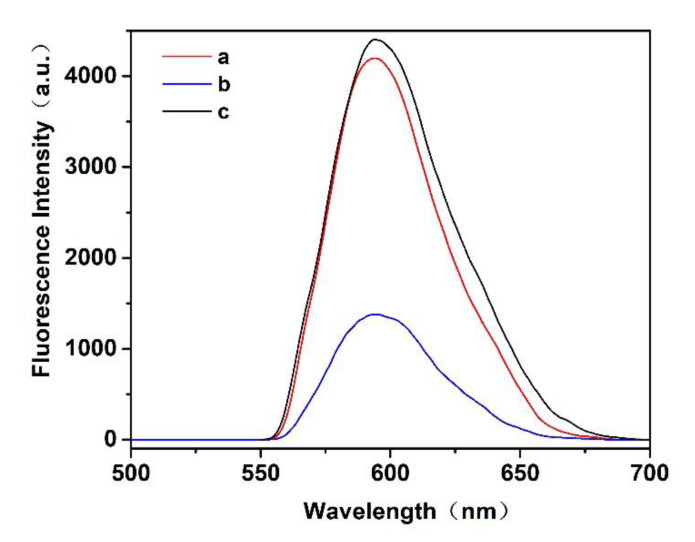
Fluorescence spectra of a MIP@QDs, b MIP@QDs rebinding of SAM, and c NIP@QDs.

The excitation and emission fluorescence spectra of the prepared MIP- and NIP@QDs were measured. The emission spectra of the MIP@QDs are symmetric, with a half-peak width of 40 nm and no obvious defect peak. The maximum fluorescence emission peak is located at 593 nm and has an orange-red fluorescence emission. Figure 2 showed that the fluorescence emission spectrum of MIP@QDs was similarly to that of NIPs. However, the MIP@QDs were significantly quenched when the MIPs selectively bind to the SAM molecules. Additionally, the fluorescence of the prepared MIP@QDs was recovered after removing the SAM molecules, which was about 95.5% of that of the NIP@QDs. This fluorescence quenching of the MIP@QDs was due to the excitation electron transfers from QDs to the template SAMs. Based on this system, a new biosensor for the selective and fluorescence detection of SAM molecules has been developed. 

**Figure 3:**
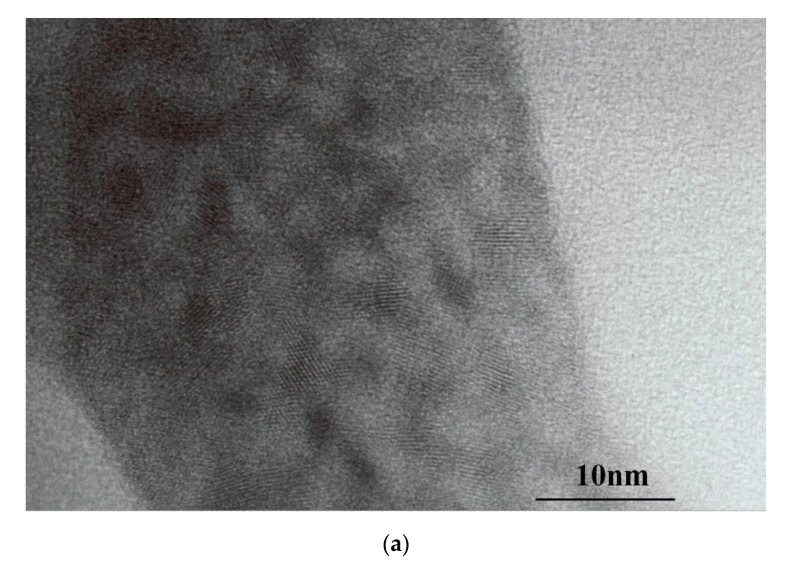

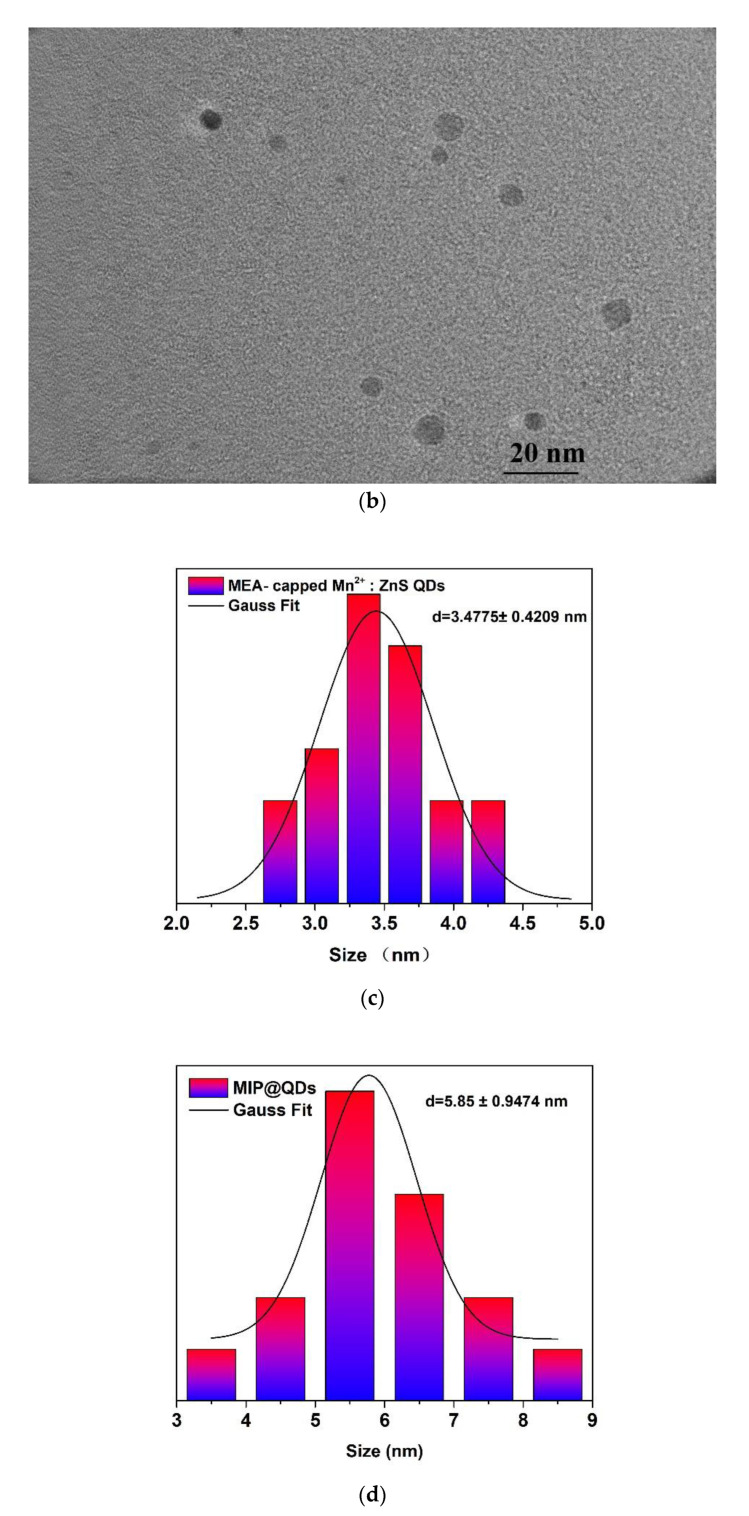
TEM images of (**a**) MEA-capped Mn^2+^: ZnS QDs, (**b**) MIP@QDs, and particle size distribution of (**c**) MEA-capped Mn^2+^: ZnS QDs and (**d**) MIP@QDs.

The morphology and size distribution of the prepared Mn^2+^: ZnS QDs and MIP@QDs were investigated using a JEM-2100F transmission electron microscope. Figure 3a showed that the prepared MEA-capped Mn^2+^: ZnS QDs has an obvious lattice structure, and the Gaussian fitting results (Figure 3c) of transmission electron microscopy showed that the particle size distribution of QDs is uniform, and the diameter size is about 3.48 ± 0.42 nm. Compared to the Mn^2+^: ZnS QDs, the MIP@QDs have larger particles sizes, which are caused by the MIP layer coated on the QDs. As shown in Figure 3b,d, the morphology of MIP@QDs is close to spherical morphology, and their diameter size is about 5.85 ± 0.95 nm.

### 3.3. Effect of the Amount of Functional Monomer

**Figure 4:**
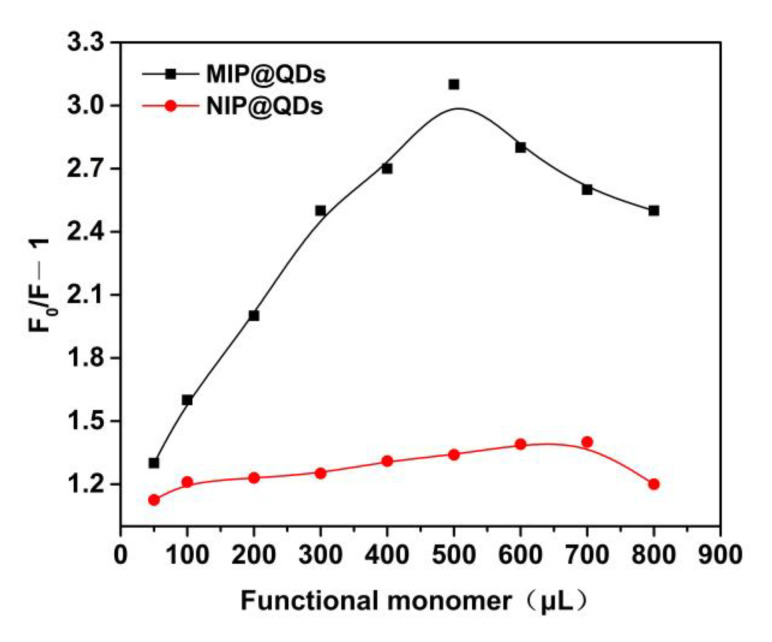
Effects of the amount of the functional monomer MAA (mg) on fluorescence quenching of MIP@QDs and NIP@QDs.

The effect of the amount of functional monomer (MAA) on the fluorescence quenching degree (△*F*) of the prepared MIP- and NIP@QDs was investigated and the results are shown in Figure 4. After binding of the template molecule (SAM), the MIP@QDs showed an increased quenching degree with the increase of the amount of the functional monomer (MAA, 0–500 μL). However, the quenching degree of the MIP@QDs was decreased with a continued increase in the usage of MAA. As a control, the amount of functional monomer did not have a significant effect on the quenching degree of the NIPs. The reason for this phenomenon is that lower usage amount of a monomer will affect the coating of the molecular imprinted polymer layer on the surface, which cannot provide enough imprinted binding sites. However, excessive use of the MAA will increase the thickness of the MIPs layer on the QDs. It is not beneficial to the binding of the SAM molecules and affects the fluorescence quenching of the MIP@QDs. The experiment result showed that the optimal amount of MAA for preparing the MIP@QDs is 500 μL.

### 3.4. Response Time 

**Figure 5:**
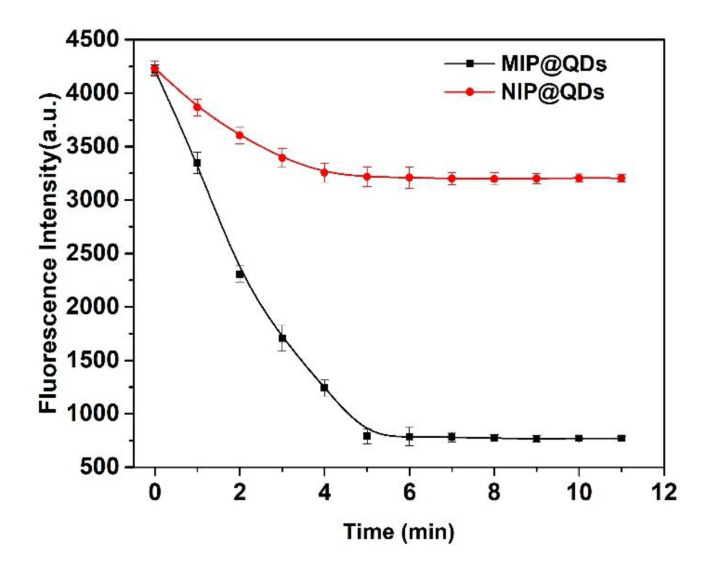
Response time of MIP@QDs and NIP@QDs.

Figure 5 showed the response time of the MIP@QDs-based sensor for SAM in the samples. When the MIPs layer on the ZnS QDs selectivity adsorbs the target molecule, the fluorescence intensity of the prepared MIP@QDs was significant decreased in the initial 0–4 min and tended to be stable after incubation for 5 min. The adsorption of the MIP@QDs to SAMs achieved equilibrium at 5 min. In contrast, the fluorescence change of the NIPs was non-significant. It is because there is no specifically imprinted site on the NIP@QDs for the target molecule, so a small number of SAM molecules were bound with the NIP@QDs by nonspecific absorption. Therefore, the quenching of the NIP@QDs was relatively slight. The results showed that the response time of the MIP@QDs for SAM was 5 min. Compared with other methods, the MIP@QDs have a short response time for SAM, without any special pretreatment steps [7,19,42].

### 3.5. Effect of pH Value on MIP@QDs

**Figure 6:**
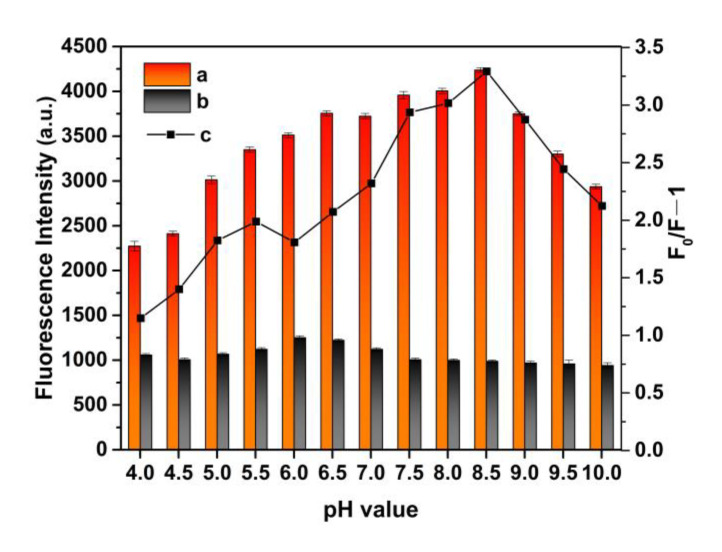
pH influence on the fluorescence intensity of MIP@QDs’ a absence and b in the presence of SAM. c Fluorescence quenching degree of the MIP@QDs. Experimental condition: buffer solution (pH 4.0–10.0, 0.02 mol L^−1^) and room temperature.

The fluorescence change of MIP@QDs in solution with different pH values was studied. As shown in Figure 6, the pH values of solution have an obvious influence on the MIP@QDs. The fluorescence intensity of the prepared polymer was decreased in the lower pH values (4.0–6.0). This is mainly because a low pH value will cause the structure of the prepared nanoparticles to change, affecting their fluorescence properties. After binding with the SAM molecule, the fluorescence quenching of molecular imprinted QDs’ nanocomposites could be observed at different pH values. The maximum quenching of molecular imprinted QDs’ nanocomposites occurred at pH 8.5. In the further experiments, pH 8.5 was selected as the optimal pH value of the solution.

### 3.6. Fluorescence Measurement of SAM 

**Figure 7:**
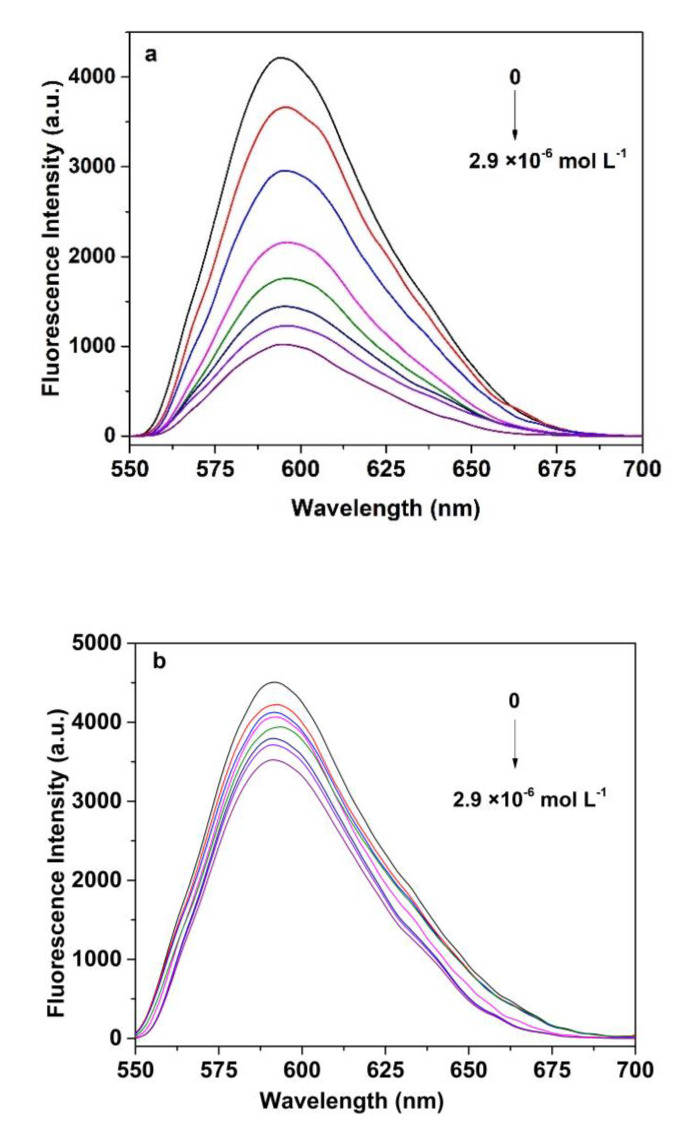

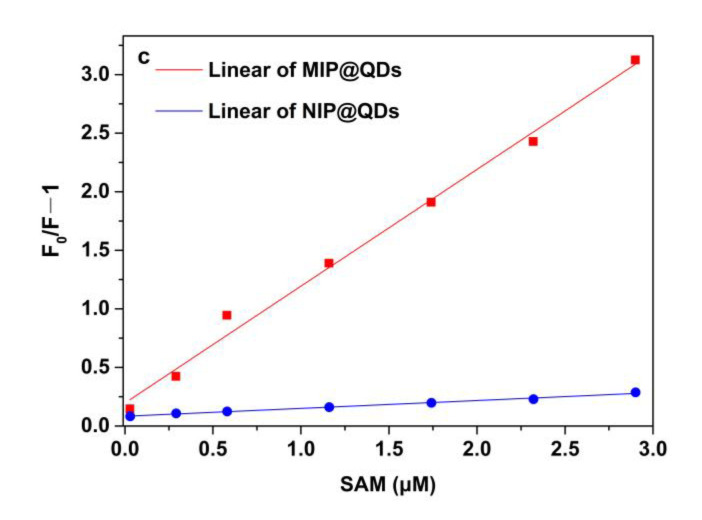
Fluorescence emission spectra of (**a**) MIP@QDs and (**b**) NIP@QDs with increasing concentrations of SAM; (**c**) the linear calibration of the fluorescence-quenching degree versus SAM concentration of MIP@QDs and NIP@QDs. Experimental conditions: SAM (0, 0.29, 2.9, 5.8, 1.16,1.74, 2.32, 2.90 μM), pH = 8.5, and room temperature.

The mechanism of detection of SAM is based on an orange fluorescence quenching of molecular imprinted QDs’ nanocomposites. Firstly, the molecular imprinted layer was specifically captured and bound to the SAM molecule. Secondly, the conductive bands’ electrons of the Mn^2+^: ZnS QDs were transferred to the SAM molecule. These electrons’ transfer generates a new nonradioactive decay pathway, which leads to the fluorescence quenching of QDs [45,47]. Figure 7 depicts the typical fluorescence quenching spectra of MIP@QDs in SAM solutions with different concentrations. As shown in Figure 7a,b, there are gradually fluorescence-quenched MIPs, with an increase in the concentration of SAM. Compared with NIPs, the emission spectra of the MIP@QDs exhibits more noticeable responses for SAM, and the quenching degree (△*F = F_0_/F* − 1) of the MIPs was 11.2 times that of the NIP@QDs. It is due to the binding sites of MIPs, which complement the SAM molecules in structure, size, shape, and intermolecular interaction. The presence of these binding sites of MIP@QDs can specifically bind more SAM molecules, resulting in more obvious fluorescence quenching. As a control, a few SAM molecules were bound with NIP@QDs by nonspecific absorption; therefore, NIP@QDs showed a lower fluorescence-quenched degree. As shown in Figure 7c, the content of SAM in the sample is proportional to the quenching degree of the MIP@QDs. There is a good linear relationship (R^2^ = 0.9916) between the fluorescence-quenching degree and the concentration of SAM in the samples, ranging from 2.90 × 10^−8^ to 2.90 × 10^−6^ mol L^−1^. The Stern–Volmer equation was *F*_0_*/F* = 0.1959 − 0.9975 [*C*]. The limit of detection (LOD) of the MIP@QDs for SAM was 3.23 × 10^−9^ mol L^−1^, following the IUPAC criterion (3*δ*/*S*).

### 3.7. Selectivity Experiments

**Figure 8:**
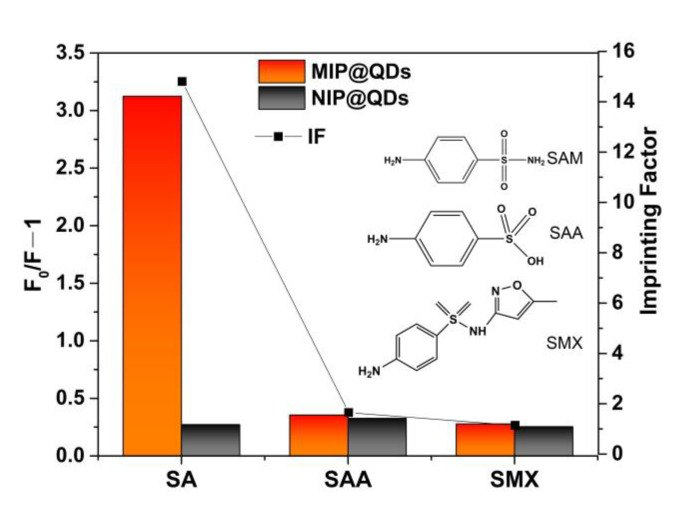
The fluorescence-quenching degree (*F*_0_*/F* − 1) and the selectivity of MIP@QDs sensor (imprinting factor) for SAM, SAA, and SMX.

In the selectivity binding test, sulfanilic acid, 4-ethylaniline, and benzenesulfonic acid were used as the comparative substrates to investigate the specificity of the MIP@QDs. As shown in Figure 8, the MIP@QDs exhibited a good recognition selectivity to the SAM molecule in the binding test. The fluorescence-quenching degree (*F*_0_*/F* − 1) on the MIPs by SAM was significant compared to the other reference molecules. In this work, the molecule imprinted polymer layer was coated on the surface of the QDs by surface molecular imprinting technology. For the MIPs, the MIP@QDs formed selective binding sites complementary to the template molecules in size, structure, shape, and functional groups during the imprinting process [34,48]. The constructed MIP@QDs have selective recognition sites for the template SAM molecule, which make the template SAM molecule able to easily access the complementary binding site of the MIP@QDs. For the NIPs, there are no recognition cavities, so the template SAM and other analytes are nonspecifically physical-adsorption bound to the NIPs. The high adsorption ability of the MIP@QDs for the template molecule (SAM) was mainly caused by the complementary imprinting sites of the MIP layer of the MNP/QDs. The rebinding experimental results showed that the constructed MIP layer provides the QDs with high selectivity for the target molecule, which makes the prepared MIP@QDs-based sensor able to efficiently select the binding target molecules through plenty of the imprinting sites. On the contrary, both SAM and its comparative substrates showed an almost similar fluorescence quenching effect on the NIP@QDs, because there are no imprinting sites that exist on the NIP@QDs. The imprinting factor (IF) for SAM was 14.8, much larger than that of the comparative substrates (SAA and SMX were 1.65 and 1.40, respectively). 

### 3.8. Application

The practical application ability of the prepared MIP@QDs was carried out using different environmental water samples from sources such as rivers, lakes, and tap water. SAM was not found in the sample at the detection limit level of 3.2 × 10^−9^ mol L^−1^ by the MIP@QDs. To further verify the accuracy of the MIP@QDs, the standard addition method was used to carry out a recovery test. As shown in Table 1, the recoveries of the MIP@QDs for SAM ranged from 96.8% to 104.3%, and the value of the RSDs was between 2.8% and 4.7% (*N* = 3). Additionally, Table 2 shows the performance of different analytical techniques for the determination of SAM. As shown in Table 2, the proposed MIP@QDs sensor not only is easy to operate and low-cost but also exhibits fast response, high selectivity, and a comparable or lower limit of detection. Thus, a convenient, fast responding, highly selective fluorescence sensor has been successfully developed for target molecule detection.

### 3.9. Recyclability and Stability 

**Figure 9:**
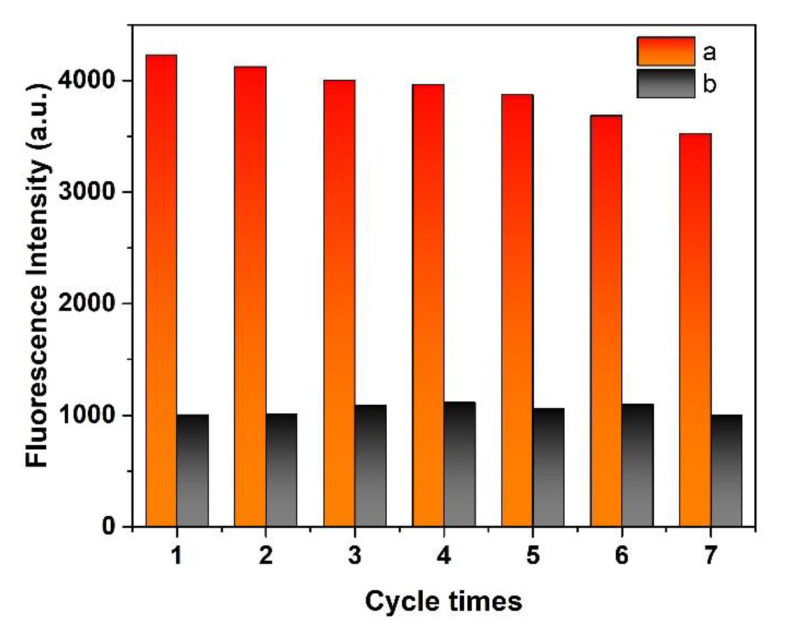
Fluorescence intensity of MIP@QDs; a removal of SAM and b rebinding of SAM.

The recyclability of the MIP@QDs was evaluated by examined the change in fluorescence intensity after a regeneration cycle test (rebind/elution). Figure 9 showed that the fluorescence of the MIP@QDs’ performance slightly decreased by seven regeneration cycle times. The fluorescence intensity decreased 8.46% after five cycles, compared with the initial reading of the sensor, indicating that the MIP@QDs have a good recyclability. Additionally, the prepared MIP@QDs had a stable fluorescence emission, with no significant change after being stored for 30 days in the dark. The experimental results showed that MIP@QDs possessed good recyclability and stability and can be used as an ideal sensor with a high practical value.

## 4. Conclusions

In this study, an advanced molecularly imprinted Mn^2+^: ZnS QDs-based sensor was successfully developed and used for the rapid and selective fluorescence sensing of SAM in water samples. The MIP@QDs-based sensor not only inherits the high selectivity of molecular imprint polymers, but also possess the excellent optical properties of Mn^2+^: ZnS QDs. The proposed method of the MIP@QDs has improved the problems of slow mass transfer and the difficult elution of traditional molecular imprinting technology and has also broadened the application range of quantum dots. The rebinding experiment results showed that the developed MIP@QDs-based sensor has the advantages of a short response time, high selectivity, and sensitivity for the target SAM molecule. In addition, the prepared sensor had a low cost, simple operation, and good stability and repeatability for the detection of SAM in water samples. These superior optical and physical merits enable the MIP@QDs-based fluorescence sensor to have promising potential in food, environmental, and biological analysis.

## Figures and Tables

**Table 1:** Detection of SAM in water samples by MIP@QDs.

Samples	Added (μM)	Measured (μM)	Recovery (%)	RSD (*n* = 3, %)
Lake water	0.1000	0.09682	96.8	3.4
	1.000	0.9733	97.3	4.2
River water	0.1000	0.1019	101.9	3.8
	1.000	1.0433	104.3	4.7
Tap water	0.1000	0.1008	100.8	2.8
	1.000	0.9887	98.9	3.2

**Table 2:** Comparison of the performance of the MIP@QD-based sensor with previously reported analytical techniques for the determination of SAM.

Recognition Element	Detection Technique	Linear Range (mol L^−1^)	LOD (mol L^−1^)	References
/	μ-SPE HPLC	5.81 × 10^−9^–1.16 × 10^−6^	1.16 × 10^−9^	[7]
/	SPE-HPLC-MS	2.91 × 10^−6^–1.16 × 10^−4^	1.16 × 10^−9^	[8]
MnO_2_ NR-IL	DPV	7.0 × 10^−8^–1.0 × 10^−4^	1.0 × 10^−8^	[11]
GCE−ND	Voltammetry	5.81 × 10^−6^–4.65 × 10^−4^	9.3 × 10^−7^	[12]
/	LDS-SD DLLME SDME-HPLC	5.81 × 10^−7^–2.90 × 10^−5^	1.12 × 10^−8^	[19]
N-Cu-MOFs	Electrochemical	1.0 × 10^−8^–5.83 × 10^−7^	3.0 × 10^−9^	[23]
rGO/GCE	Amperometric	1.0 × 10^−5^–5.0 × 10^−5^	2.3 × 10^−6^	[26]
MIPs/QDs@SiO_2_	FL sensing	2.0 × 10^−6^–3.0 × 10^−5^	1.7 × 10^−7^	[42]
MIP@QDs	FL sensing	2.9 × 10^−8^–2.90 × 10^−6^	3.2 × 10^−9^	This work

SPE: solid-phase extraction; HPLC: high-performance liquid chromatography; MS: mass spectrometry; NR: nanorods; IL: ionic liquid; DPV: differential pulse voltammetry; ND: nanodiamond; GCE: glassy carbon electrode; LDS-SD-DLLME-SDME: low-density solvent-based solvent-demulsication dispersive liquid–liquid microextraction single-drop microextraction; rGO/GCE: reduced graphene oxide glassy carbon electrode; Fe_3_O_4_@COF@Au-β-CD: β-Cyclodextrin-functionalized magnetic covalent organic framework; MSPE: magnetic solid-phase extraction; MIP: molecular imprinted polymer; QDs: quantum dot.

## Data Availability

All the data generated by this research are included in the article.

## Abbreviations

MIP, molecular imprinted polymers; QDs, quantum dots; Mn^2+^: ZnS QDs manganese-doped zinc sulfide quantum dots; MIP@QDs, molecule imprinted quantum dots; NIP@QDs, non-imprinted polymers; SMX, sulfanilamide; SMIT, surface molecular imprinting technique; RSD, relative standard deviation; LOD, limit of detection.
